# Supplementary material for: Membrane reshaping by micrometric curvature sensitive septin filaments
Source: Nat Commun. 2019 Jan 24;10:420. doi: 10.1038/s41467-019-08344-5 (PMC6345803; doi:10.1038/s41467-019-08344-5)
Supplement: Supplementary file 1 — Supplementary Information [file 41467_2019_8344_MOESM1_ESM.pdf]

Supplementary information

Membrane reshaping by micrometric curvature sensitive septin filaments

Beber et al.

Supplementary figure 1.

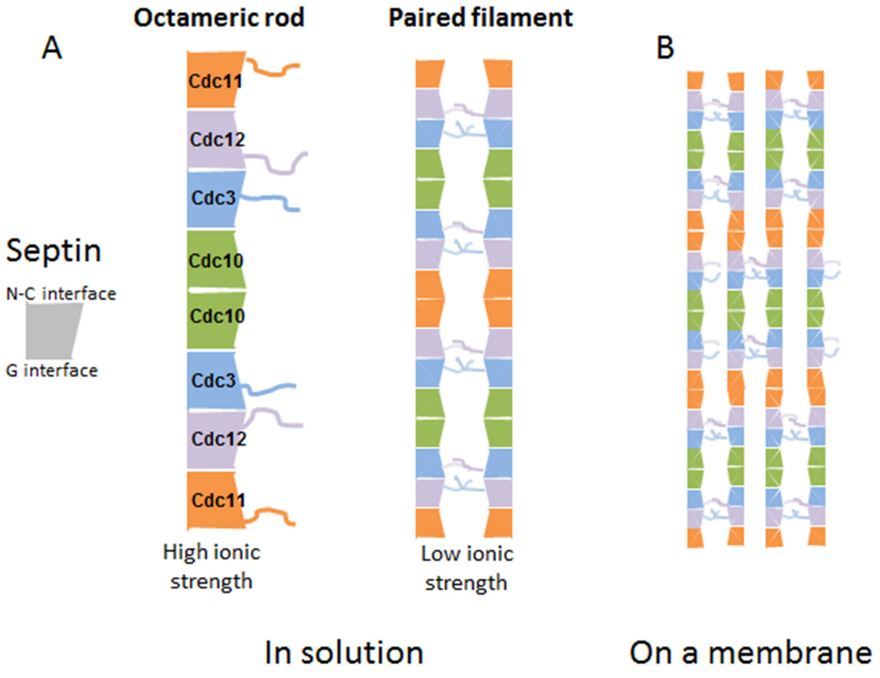

A. Schematic representation of an octameric Septin rod in solution. Septins are arranged in a palindromic fashion and self-assemble into non polar paired filaments (right).

B. Arrangement of Septin filaments bound to a membrane. To maintain a regular spacing in between filaments, imposed by coiled coils, the octamers might be oriented differently within the same filament.

Supplementary figure 2.

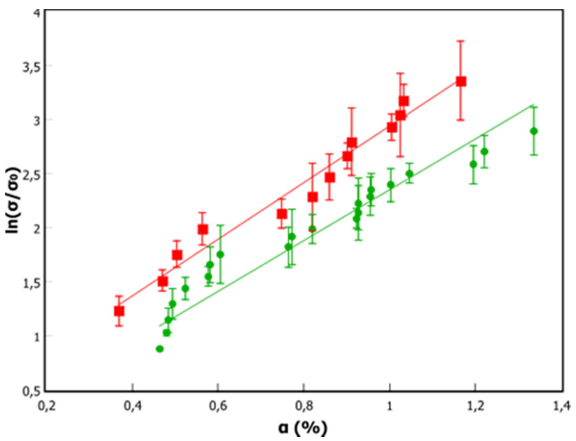

Measurement of the mechanical properties of a single GUV at low ($\sigma < 0.5$ mN.m^{-1}) tension regime without (red) and with (green) septins. The graph shows the logarithm of the applied tension versus the relative increase in area. The slope allows us to extract a value of bending modulus of 9.4 ± 0.5 K_bT (left) and 9.7 ± 0.5 K_bT (right). Error bars are s.d.

Supplementary figure 3.

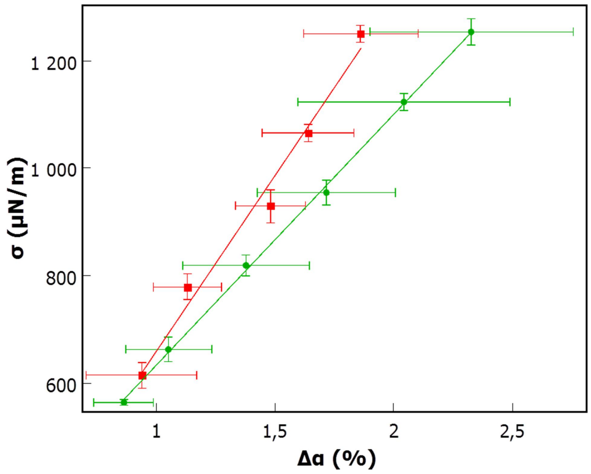

Applied tension versus area expansion at high tension (>0.5 mN/m). The red curve corresponds to the control and the green curve to the septin-coated experiments. The values extracted from the slopes are $K_{control} = 65 \pm 9$ mN/m and $K_{septins} = 42 \pm 8$ mN/m. Error bars are s.d.

Supplementary figure 4.

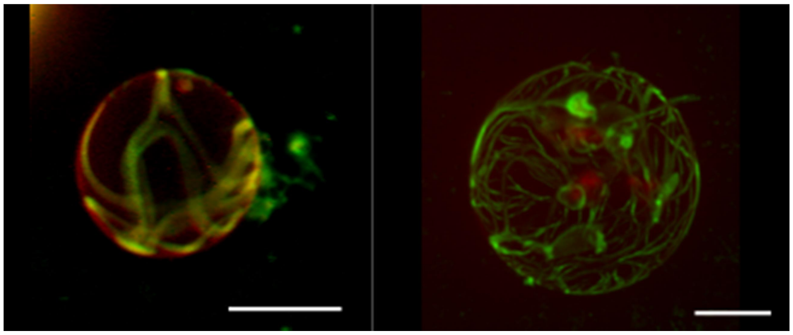

Top: 3D reconstruction of confocal images of GUVs in a solution of 200 nM septins. Lipids are in red and septins in green. Septins can form a network of bundles on the vesicle.

Scale bars = 10µm.

Supplementary figure 5

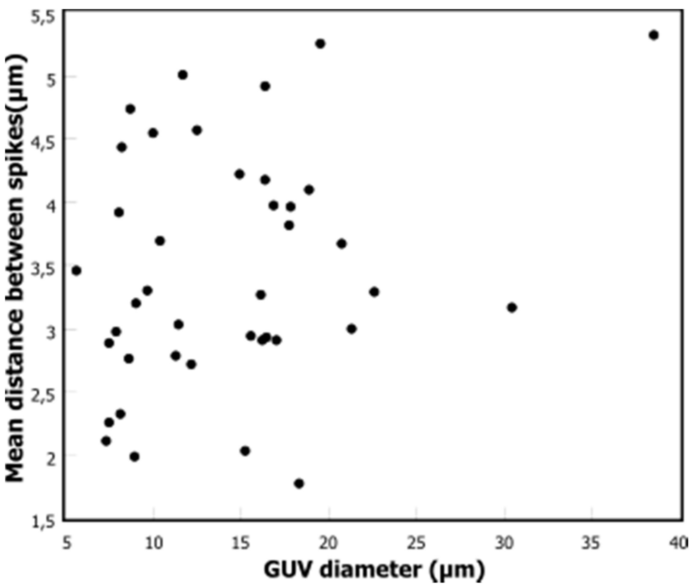

Mean distance between spikes measured for individual GUVs as a function of the GUV diameter. The distance appears to be independent of the GUV diameter.

Supplementary figure 6

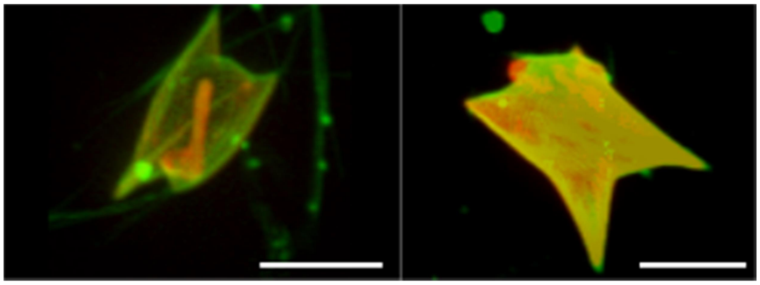

3D reconstruction of confocal images of GUVs in a solution of 200nM after 24h of incubation. Large deformations (spikes) are visible. Scale bars are 1µm.

Supplementary figure 7

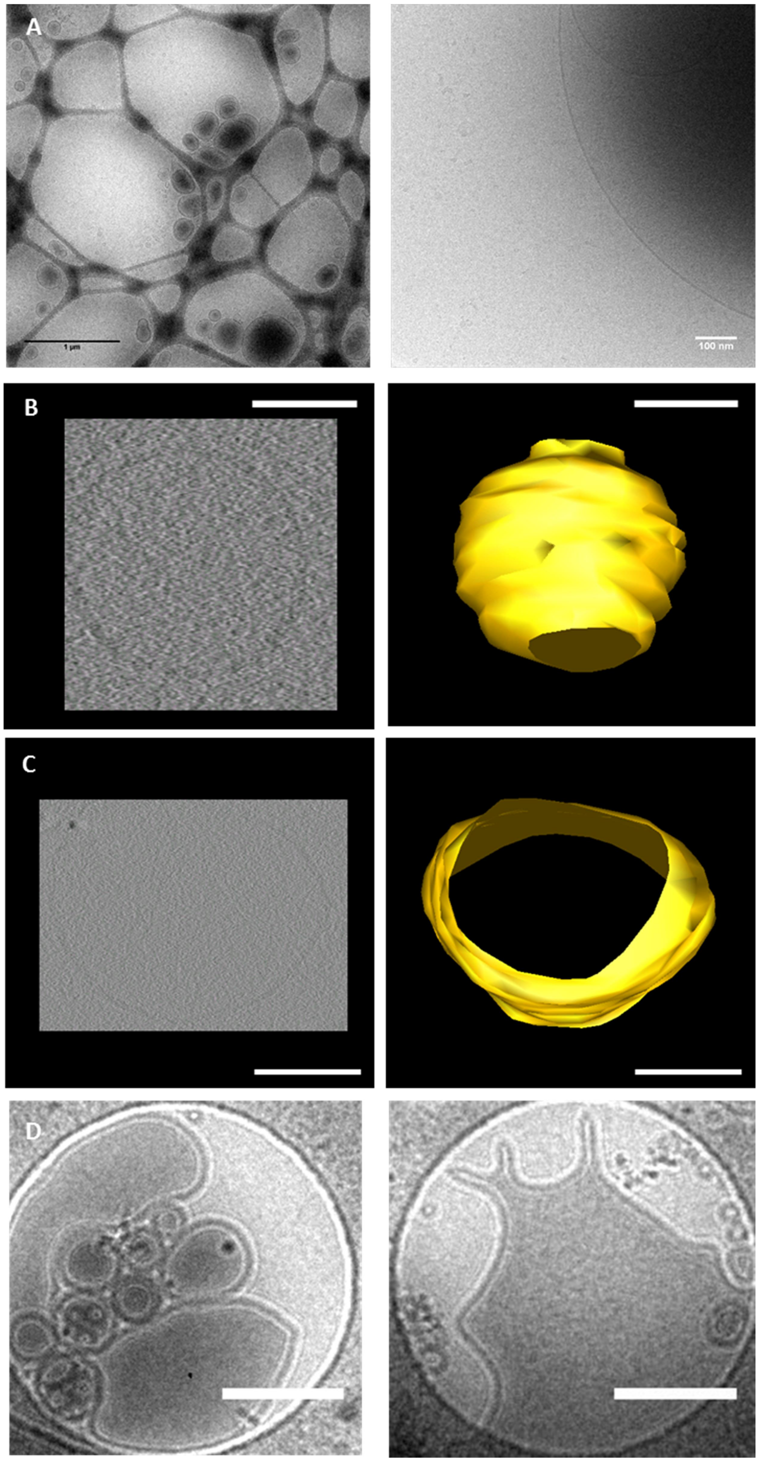

A.Control image of naked LUVs at low magnification (left, scale bar: 1µm) and higher magnification (right, scale bar: 100 nm). B and C. slices in tomograms carried out on control samples (left). The membrane segmentation is shown in yellow (right). C: scale bar 50 nm, D: scale bar 100 nm. D. Enlarged inserts from figure 3.A, right panel. Scale bar: 500 nm.

Supplementary figure 8

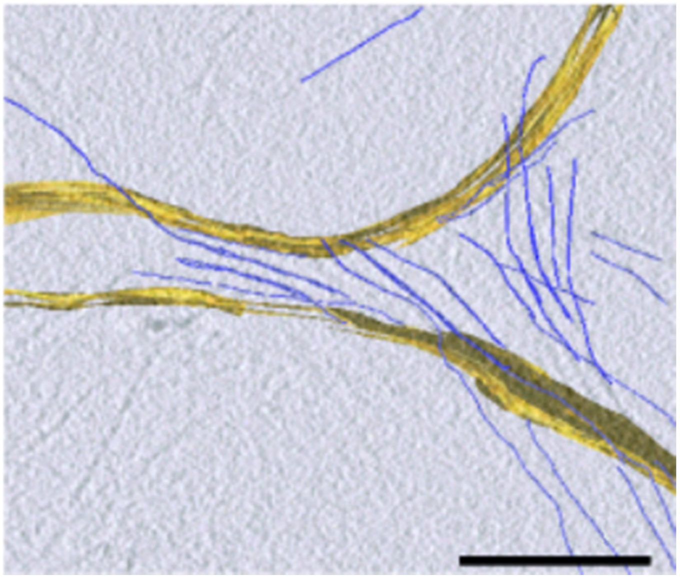

Example of a slice within a tomogram where filaments arrange in the vicinity of a protrusion. Scale bar: 200 nm.

Supplementary figure 9

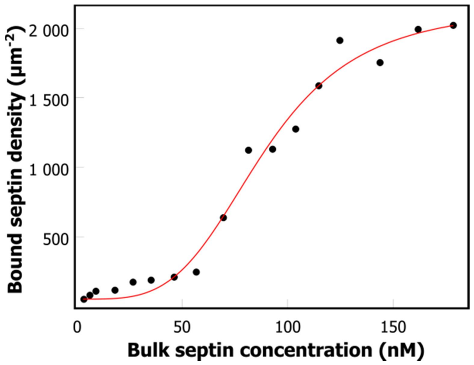

Bound septin density on a flat supported lipid bilayer as a function of bulk septin concentration (black dots).

The Kd value (88 ± 6 nM) was extracted by fitting with a Hill equation (red curve).

Supplementary figure 10

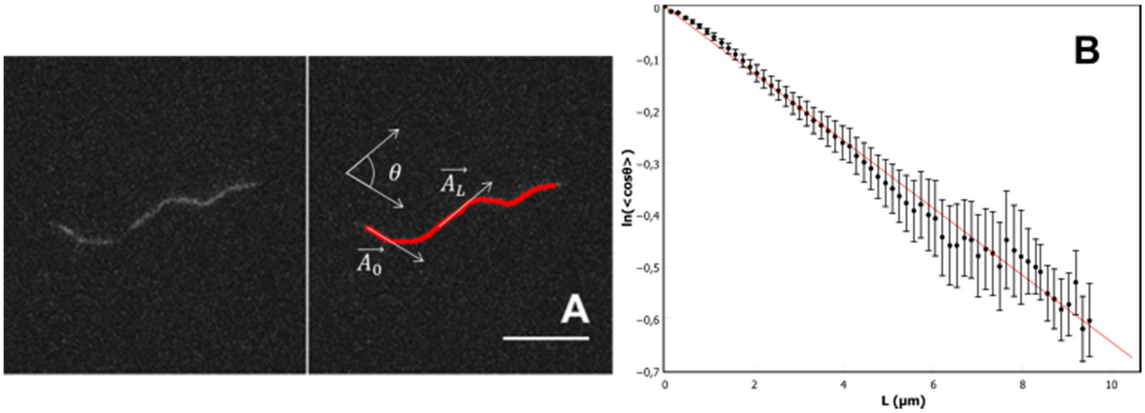

A) TIRF image of a septin filament on a surface passivated with PLL-PEG (left). Image of the same filament tracked by the jFilament plug-in (right). Scale bar 10 µm.

B) Mean angle of unit vectors tangential to the septin filaments as a function of arc length. The slope gives the persistence length of double septin filaments. Lp = 7.8 ± 1.1 µm. Error bars are s.d.

Supplementary figure 11

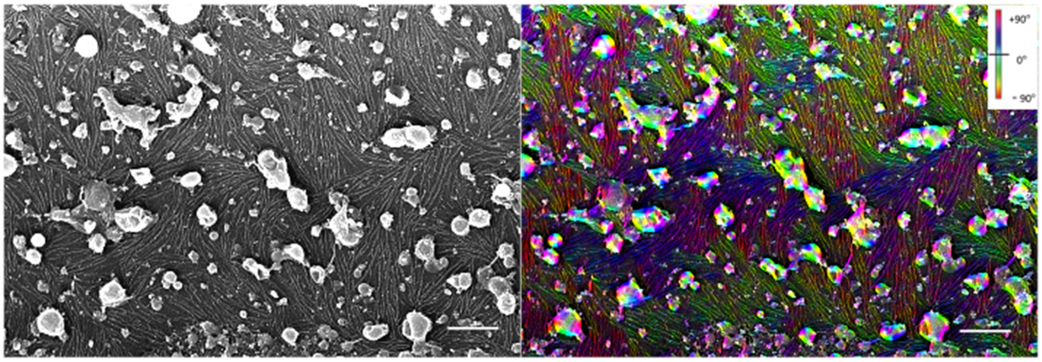

SEM image of septin filaments on a flat supported lipid bilayer (left). Same image colored with the OrientationJ plug-in (right). Scale bar 200 nm. Filaments show no preferential orientation.

Supplementary figure 12

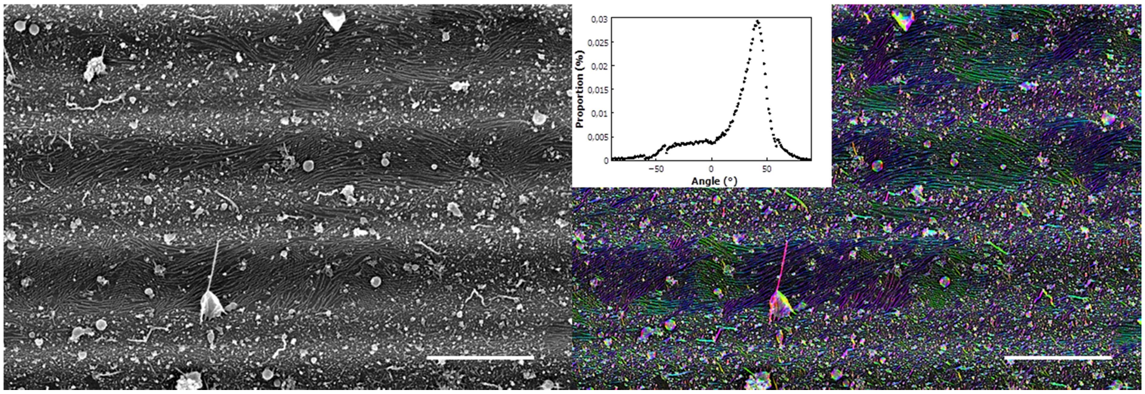

SEM image of septin filaments on a curved supported lipid bilayer, Cmax = 1.6 µm-1 (left). Same image colorized by the OrientationJ plug-in, 0° orientation corresponds to the axis of null curvature (right). Graph shows the angular distribution of pixels which displays a peak at about 40°. Scale bar 1µm. Filaments show a less organized distribution at this curvature.

Supplementary figure 13

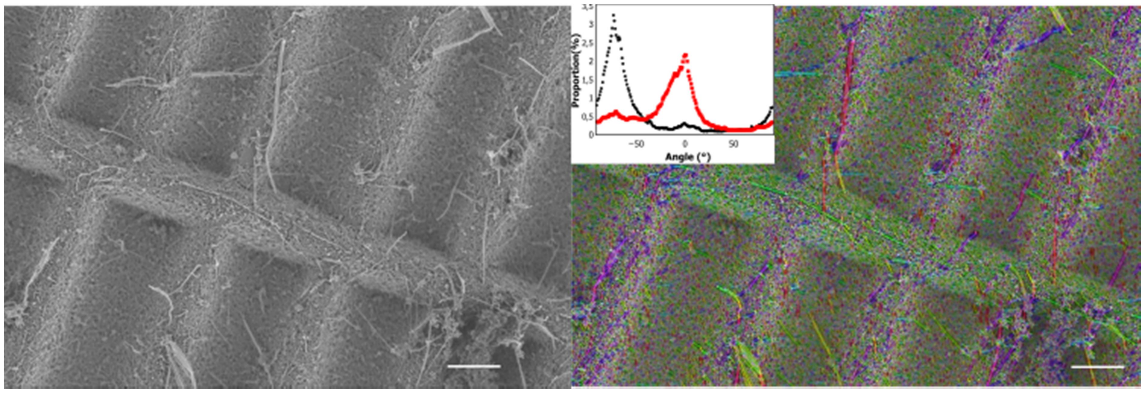

SEM image of septin filaments on a patterned substrate after 24h of incubation (left) Cmax = 3µm-1. Same image colorized with the OrientationJ plug-in (right). Graph shows the angular distribution of pixels. 0° orientation corresponds to the axis of null curvature for negative (black) and positive (red) curvatures. Scale bar 1µm.

Supplementary figure 14

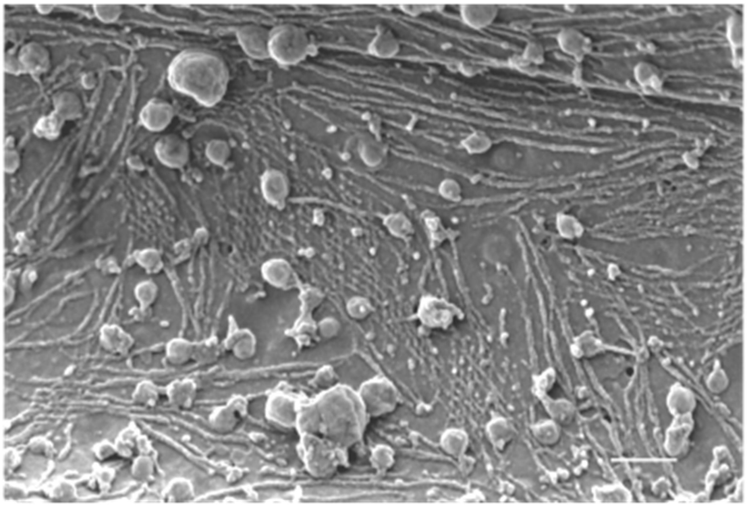

SEM image of septin filaments on a patterned surface (Cmax = 3µm-1) showing crossed filaments. Scale bar 200 nm.
