# Supplementary material for: Membrane reshaping by micrometric curvature sensitive septin filaments
Source: Nat Commun. 2019 Jan 24;10:420. doi: 10.1038/s41467-019-08344-5 (PMC6345803; doi:10.1038/s41467-019-08344-5)
Supplement: Supplementary file 7 — Description of Additional Supplementary Files [file 41467_2019_8344_MOESM7_ESM.docx]

**File Title:** Supplementary movie 1
**Description:** Cryo-tomogram of a vesicle and protrusion deformed by Septin filaments. Each consecutive image represents one slice (in z) within the 3D reconstruction. The segmentation represents the membranes in yellow and the Septin filaments in blue. Scale bar 200 nm.

**File Title:** Supplementary movie 2
**Description:** Cryo-tomogram of a vesicle covered by a parallel array of Septin filaments. Each consecutive image represents one slice (in z) within the 3D reconstruction. The segmentation represents the membranes in yellow and the Septin filaments in blue. The width of the whole image is 852 nm.

**File Title:** Supplementary movie 3
**Description:** Cryo-tomogram of a naked vesicle. Each consecutive image represents one slice (in z) within the 3D reconstruction. The segmentation represents the membranes in yellow. Scale bar 50 nm.

**File Title:** Supplementary movie 4
**Description:** Cryo-tomogram of a naked vesicle. Each consecutive image represents one slice (in z) within the 3D reconstruction. The segmentation represents the membranes in yellow. Scale bar 100 nm.
